# The Role of Reduced Oxygen Supply and Transcription Factors cJUN and CREB in Progesterone Production during the Corpus Luteum Rescue in Gilts

**DOI:** 10.3390/ani12202855

**Published:** 2022-10-20

**Authors:** Emilia Przygrodzka, Kamil Myszczynski, Jan Klos, Adam J. Ziecik

**Affiliations:** Department of Hormonal Action Mechanisms, Institute of Animal Reproduction and Food Research, Polish Academy of Sciences, Tuwima 10, 10-748 Olsztyn, Poland

**Keywords:** corpus luteum, hypoxia, progesterone, hypoxia inducible factor-1, *STAR*

## Abstract

**Simple Summary:**

Appropriate function of the corpus luteum and progesterone secretion are fundamental for the establishment and maintenance of pregnancy. Still, there are gaps in the knowledge of mechanisms regulating the maintenance of steroidogenic machinery of porcine corpus luteum during early pregnancy. In the present study, we analyzed the in vitro effects of decreasing oxygen concentration on progesterone production, expression of hypoxia inducible factor-1 subunit alpha (*HIF-1A*) and expression of genes encoding key stimulators of steroidogenesis, *STAR* and *VEGFA*, in the luteal tissue collected from cyclic and early pregnant gilts. In vitro experiments indicate that: (1) progesterone production and genes expression are modulated by decreasing oxygen concentrations and (2) luteal tissue of cyclic and pregnant gilts is similarly sensitive to reduction of oxygen. We also determined the mRNA and protein expression of HIF-1α, *STAR*, and *VEGFA* as well as transcription factors-cJUN and CREB, which are known regulators of *STAR* expression, in the luteal tissue of cyclic and pregnant gilts ex vivo. The relatively high level of *STAR*, phosphorylated forms of cJUN and CREB and progesterone concentration in the luteal tissue of early pregnant gilts suggests the role of these transcription factors in extension of luteal function. This regulation may be important in supporting luteal rescue during early pregnancy to maintain the production of progesterone. A reduced concentration of oxygen could be involved in the process of corpora lutea regression in gilts.

**Abstract:**

The corpus luteum plays a fundamental role in regulating reproduction via progesterone production. Still, there is little data on factors regulating the maintenance of luteal function during early pregnancy in gilts. Previous studies emphasize the role of hypoxia and HIF-1 in the regulation of steroidogenic and angiogenic genes expression and progesterone production by ovarian cells. Using the corpus luteum of cyclic and early pregnant gilts we analyzed: (1) the in vitro effects of reduced oxygen tension on progesterone production and mRNA expression of *HIF1A* and luteal function regulators, *STAR* and *VEGFA*; (2) the ex vivo profiles of mRNA and protein expression of HIF-1α, *STAR*, *VEGFA* and transcription factors-cJUN and CREB, regulating *STAR* expression, in the corpus luteum of cyclic and pregnant gilts. The synthesis of progesterone was gradually inhibited in cyclic or pregnant gilt luteal tissue (on day 13 of cycle or pregnancy) incubated in a decreasing concentration−20%, 10%, and 3% of oxygen (O_2_). Luteal tissues of pregnant gilts produced trace amounts of progesterone in 10% O_2_, which was similar to cyclic gilts in 3% O_2_. *HIF1A* expression increased after 24 h of incubation in tissues of cyclic gilts in 3% vs. 20% O_2_ (*p* < 0.01), whereas levels of *STAR* and *VEGFA* increased significantly in cyclic and pregnant gilt tissues incubated in 10% and 3% vs. 20% O_2_. The ex vivo mRNA expression of *HIF1A* and *VEGFA* was elevated (*p* < 0.001) on day 14 vs. day 12 of pregnancy. The protein expression of HIF-1 and *VEGFA* increased (*p* < 0.001), whereas the level of *STAR* (mRNA and protein) and progesterone dropped (*p* < 0.001) on day 14 of the estrous cycle vs. a parallel day of pregnancy and/or day 12 of the estrous cycle. The content of phosphorylated cJUN and CREB was elevated (*p* < 0.01) in the luteal tissue on day 12 or 14 of pregnancy vs. parallel days of the estrous cycle. These increases of phosphorylated cJUN and CREB may be involved in *STAR* expression in the luteal tissue during early pregnancy in gilts.

## 1. Introduction

The corpus luteum is an endocrine gland producing progesterone, a steroid hormone crucial for controlling the estrous cycle and preparing the uterine environment for embryo implantation and development. Thus, adequate production of progesterone is fundamental for the establishment and maintenance of pregnancy. In the absence of conceptuses in the uterus, the corpus luteum undergoes regression, reflected by disrupted production of progesterone (functional luteolysis) and followed by luteal cell death (structural luteolysis) [1,2]. In contrast, the presence of live embryos in the uterus prolongs luteal function due to the luteotropic and/or antiluteolytic action of signals from conceptuses in most mammalian species [1,2].

The main substrate for progesterone synthesis is cholesterol, which can be obtained from low- and high-density lipoproteins, lipid droplets or can be de novo synthesized by luteal cells [3]. Once inside the cell, cholesterol is transported to mitochondria by the steroidogenic acute regulatory protein (*STAR*). Furthermore, cholesterol is converted to pregnenolone via the cholesterol side-chain cleavage enzyme (CYP11A1) followed by conversion to progesterone by 3β-hydroxysteroid dehydrogenase (HSD3B) [3,4]. In the ovary, *STAR* is recognized as the most important regulator of progesterone production [5,6]. The major pathway regulating *STAR* expression and activation is protein kinase A (PKA) triggered mainly by luteinizing hormone (LH), a gonadotropin regulating formation, development, and maintenance of the corpus luteum in domestic animals such as pigs or cows [7,8,9]. The expression of *STAR* is controlled by transcription factors sharing the same binding site in *STAR* promoter such as CREB (cAMP response element binding protein), and cJUN (member of AP-1 family), as well as recently identified hypoxia inducible factor (HIF)-1 [9,10,11]. 

Hypoxia and HIF-1 were recognized as the vital factors regulating corpus luteum development and regression [12,13,14,15,16,17,18,19,20]. HIF-1 is formed by HIF-1α and constitutively expressed HIF-1β. Hypoxic conditions induced by a low concentration of oxygen (O_2_) prevent degradation of HIF-1α via the ubiquitin–proteasome pathway leading to its stabilization in the cytoplasm. In contrast, under a high concentration of O_2_, HIF-1α is degraded in the proteasome. The high O_2_ maintains HIF-1α at a low level in cells and consequently precludes the activation of HIF-1 and its downstream action. In hypoxic conditions, HIF-1α protein content, further HIF-1 formation, and transcription of HIF-1 targeted genes increase [21]. On the other hand, the HIF-1 level can also be regulated in an O_2_-independent way [22,23]. HIF-1 significantly increases in mouse granulosa cells at the time of ovulation and its abundance is regulated by the limited supply of O_2_ due to impaired development of the vasculature system as well as stimulatory effects of gonadotropins and growth factors [24]. Hypoxic conditions enhance progesterone production and expression of vascular endothelial growth factor (*VEGFA*) and *STAR* in the primary and luteinized bovine granulosa cells, which indicate the essential role of hypoxia and HIF-1 in the secretory function and development of the vasculature system during luteal formation [11,13,14,15,17,18,20,25,26]. In contrast, incubation of luteal cells in low O_2_ concentrations significantly inhibits basal- and LH/hCG-stimulated progesterone production as well as induces apoptosis [14,15,16,17,18,20]. 

To date, the effects of hypoxia on steroidogenesis have been extensively studied in granulosa, luteal, or luteinized granulosa cells using mouse, human or cow models [13,14,15,16,17,18,20,22,23,24,26,27]. However, the role of reduced concentrations of O_2_ on basal progesterone production and/or hypoxia-induced molecular changes in the luteal tissue slices has received little attention. It also remains unknown which transcription factors are the key regulators of *STAR* expression and progesterone production in the corpus luteum of gilts during early pregnancy. In the present study, we determined: (1) the in vitro effects of decreasing concentrations of O_2_ on the basal production of progesterone and expression of *HIF1A*, *STAR* and *VEGFA* in the cultured luteal tissue slices of cyclic and early pregnant gilts during the period of corpora lutea regression or rescue, respectively; and (2) the mRNA and protein levels of HIF-1α, *STAR*, *VEGFA* and phosphorylated forms of cJUN (Ser73) and CREB (Ser133) in the corpus luteum of cyclic and early pregnant gilts ex vivo. 

## 2. Materials and Methods

### 2.1. Animal Preparation and Ovary Collection

Procedures performed on animals were conducted in accordance with the national guidelines for the care of agricultural animals. Crossbred gilts (*n* = 42), ages 6 to 8 mos., after exhibiting two estruses, were randomly divided into two groups: cyclic and pregnant. Gilts in the pregnant group were inseminated on Day 0 of the estrous cycle as previously described [19]. Pregnancy was confirmed based on the presence and morphology of conceptuses, as previously described [19]. For in vitro experiments, gilts (*n* = 14; 7 per day of the estrous cycle or pregnancy) were slaughtered on day 13 of the estrous cycle or pregnancy, ovaries were immediately collected (within 15 min), and transported on ice in Krebs–Ringer Buffer to the laboratory in order to prepare luteal slices as previously described [19,28]. Two slices (around the fourth or fifth scrap from each corpus luteum) of the corpus luteum representing technical replicates from each animal were cultured in each in vitro condition. For ex vivo experiments, the gilts (*n* = 28) were slaughtered on day 12 and 14 of the estrous cycle or pregnancy, ovaries were immediately collected and corpora luteal were immediately snap-frozen and kept in −80 °C for further analysis of gene or protein expression and measurement of progesterone concentration. 

### 2.2. Culture of Tissue Slices in Different Concentrations of Oxygen

Corpora lutea were cut into thin slices (thickness 180 μm) using a special microtome designed for processing unfixed tissue (Krumdick Tissue Slicer). Subsequently, tissue slices were preincubated for 2 h in M199 (Sigma-Aldrich, Saint Louise, MO, USA) supplemented with 0.1% BSA (ICN Biomedicals, Inc., Costa Mesa, CA, USA) and antibiotics (penicillin and streptomycin; Sigma-Aldrich). Then, the media was removed, slices were washed with warm PBS and fresh M199 with 0.1% BSA, and antibiotics were added into each well. The slices were incubated in (1) a high concentration of O_2_ (20% O_2_, 5% CO_2_, 75% N_2_) as a control and (2) reduced concentrations of O_2_ (10% or 3% O_2_, 5% CO_2_, 85% or 92% N_2_, respectively) in 37 °C for 6 and 24 h. After incubation, media samples were collected to determine progesterone concentrations. Luteal slices were snap-frozen and kept in −80 °C for further analysis of gene expression.

### 2.3. Bioinformatic Analysis

The porcine *HIF1A*, *STAR*, and *VEGFA* were scanned for matches to hypoxia response elements (HREs) [(A/G)CGTG]. First, the DNA sequences of a promoter (5000 bp upstream) with 5′UTR regions of gene transcripts of *Sus scrofa* (Sscrofa11.1 assembly) were obtained via the University of California, Santa Cruz (UCSC) Table Browser [29]. Afterward, the HRE motifs were identified within the nucleotide sequences using FIMO within MEME Suite 5.1.0 (no mismatches were allowed, *p*-value set to 0.001).

### 2.4. RNA Isolation and qPCR

Isolation of RNA was performed using a RNeasy Mini Kit according to the manufacturer’s protocol (Qiagen, Hilden, Germany). Frozen luteal tissue slices (two pulled technical replicates per animal) or pieces of the corpus luteum were placed in Lysing Matrix D tubes (MP Biomedicals Inc., Solon, OH, USA) filled with lysis buffer and homogenized in FastPrep-24 homogenizer (MP Biomedicals Inc., Solon, OH, USA). After homogenization, the supernatant was collected, and each sample was mixed with ethanol and transferred to a spin column. Next, samples were centrifuged and washed with buffers provided in the kit. RNA was treated with DNase I and eluted with nuclease-free water. The quantity and purity of RNA were determined using a Nanodrop Spectrometer (Agilent Technologies, Waldbronn, Germany). RNA samples with RIN above 6 were used for further qPCR analysis.

Afterward, RNA (15 ng) was reverse transcribed and amplified using a TaqMan RNA- to-Ct 1-step kit (Thermofisher Scientific, Waltham, MA, USA). The expression of genes was analyzed using one-step quantitative real-time PCR (qPCR) with a ABI 7900 HT Sequence Detection System (Applied Biosystems) and following TaqMan Assays: *HIF1A* (Ss03390447_m1), *STAR* (Ss03381250_u1), *VEGFA* (Ss03393993_m1), *CREB1* (Ss03386122_u1), *cJUN* (Ss03382061) and *GAPDH* (Ss03375435_u1). Each qPCR reaction was performed in duplicates on 384-well plates. GAPDH was selected as the reference gene accordingly to previous studies [30]. The expression of GAPDH did not change after incubation in decreasing concentrations of O_2_. Real-time PCR Mainer Software was used to estimate the mean PCR amplification efficiency for each gene [31].

### 2.5. Tissue Homogenization 

The homogenization of luteal tissue was performed as previously described [19,32]. Luteal tissue (100 mg) was mechanically homogenized in 5% trichloroacetic acid. The lysate was centrifuged at 10,000× *g* for 5 min, and supernatant was stored at −20 °C.

### 2.6. Progesterone Assay

The concentration of progesterone in media samples and tissue homogenates was assessed using the RIA method and commercially available kits (DIAsource ImmunoAssays S.A., Ottignies-Louvain-la-Neuve, Belgium) according to the manufacturers’ protocols, as previously described [19,28,33]. The sensitivity of the assay was 0.19 pg/mL. The intra-assay coefficient of variation for progesterone assay was 4.6%. 

### 2.7. Protein Extraction and Western Blotting

Protein extraction and western blot analysis were performed as previously described [19,34]. Small pieces of the corpus luteum were placed in homogenization buffer (50 mM Tris-HCl, pH 7.4; 10 mM EDTA; 150 mM NaCl; 1% Triton X-100; and 100 mM Protease inhibitor cocktail-PMSF) and then mechanically homogenized. Afterward, samples were centrifuged at 800× *g* for 10 min at 4 °C. The protein concentrations were measured using Bradford method. Further protein samples were separated on TGX Stain-Free 10% gels (Bio-Rad, Hercules, CA, USA). Before the transfer of protein onto the polyvinylidene difluoride membrane (Sigma-Aldrich), the TGX Stain-Free gels were activated to obtain the total content of loaded protein, according to the manufacturer’s instructions. After wet transfer, membranes were incubated for 1 h at room temperature in 5% nonfat dry milk to block unspecific binding sites. Next, membranes were incubated overnight at 4 °C with primary polyclonal rabbit antibodies diluted in TBS-T Buffer (50-mM Tris-HCl, pH 7.4; 150-mM NaCl; and 0.1% Tween 20) as follows: anti-phospho-cJUN (Ser73) (1:1000; 3270; Cell Signaling Technology; Beverly, MA, USA), anti-cJUN (1:1000; 9165; Cell Signaling Technology), anti-CREB (1:1000; 4820; Cell Signaling Technology), anti-phospho-CREB (Ser133) (1:1000, 9198; Cell Signaling Technology), anti-HIF1α (1:1000, 14179; Cell Signaling Technology). The next day, the membranes were washed in TBS-T buffer and placed for 1 h in secondary antirabbit HRP-conjugated antibodies (1:20,000; Sigma–Aldrich). Immune complexes were visualized using Clarity ECL substrate (Bio-Rad) according to the manufacturer’s protocol and developed in the ChemiDoc™ Touch Imaging System (Bio-Rad). The optical density of the protein bands detected on the membranes, and the intensity of the protein bands on the TGX Stain-Free gels was analyzed using Image Lab 6 software (Bio-Rad). The abundance of tested proteins was quantified and normalized to the total protein content in each equivalent lane or β-actin (ACTB; for HIF-1α). 

### 2.8. Statistical Analysis

All statistical analyses were done using one- or two-way ANOVA followed by the Tukey or Bonferroni post hoc test, respectively. Numerical data are expressed as mean ± standard error of the mean (SEM), and results were considered statistically significant at *p* < 0.05 (Prism version 8.0 software; GraphPad Software, Inc., La Jolla, CA, USA). 

## 3. Results

### 3.1. Decreasing the Concentration of Oxygen Reduces Progesterone Synthesis by the Luteal Tissue of Cyclic and Pregnant Gilts In Vitro

**Figure 1:**
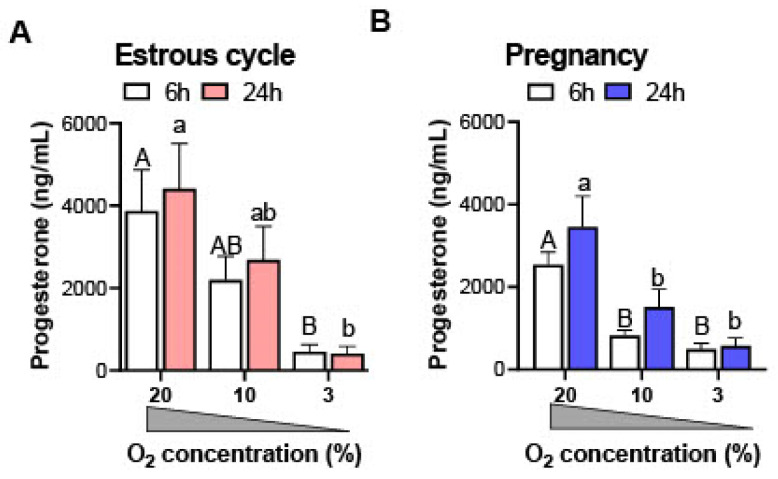
The concentration of progesterone in media samples collected after incubation of luteal tissue obtained from cyclic (day 13 of the estrous cycle; (**A**)) and early pregnant gilts (day 13 of pregnancy; (**B**)) in different concentrations of O_2_ (20, 10, and 3% O_2_) for 6 and 24 h. Data are presented as mean ± SEM (*n* = 5–7 per reproductive status). The analysis was performed using a two-way ANOVA with Bonferroni post-hoc test. Capital and small letters reflect significant differences between different concentrations of O_2_ for 6 and 24 h incubations, respectively.

In order to determine the effects of decreasing O_2_ concentrations on the secretory function of the luteal tissue, tissue slices were prepared on day 13 of the estrous cycle or pregnancy and incubated at high (20%) and reduced (10% and 3%) O_2_ concentrations, similar to previous studies [13,14,15,16,20]. Luteal slices from cyclic and pregnant animals incubated in 20% O_2_ produced the highest amount of progesterone (Figure 1A,B). A decreasing concentration of O_2_ gradually inhibited the synthesis of progesterone by the luteal tissues of cyclic or pregnant animals after 6 and 24 h incubations. In the luteal tissues of cyclic gilts, the concentration of progesterone significantly dropped after 6 and 24 h incubations in 3% O_2_ vs. 20% O_2_ (by 88% and 91%, respectively; *p* < 0.01; Figure 1A). In luteal slices of pregnant gilts, the production of progesterone was markedly suppressed (*p* < 0.01) after 6 and 24 h incubations in 10% O_2_ (by 68% and 56%, respectively), 3% O_2_ (81% and 84%, respectively), and 20% O_2_ (Figure 1B).

### 3.2. Decreasing the Concentration of Oxygen Changes the mRNA Expression of HIF1A, STAR, and VEGFA in the Luteal Tissue of Cyclic and Pregnant Gilts In Vitro

**Figure 2:**
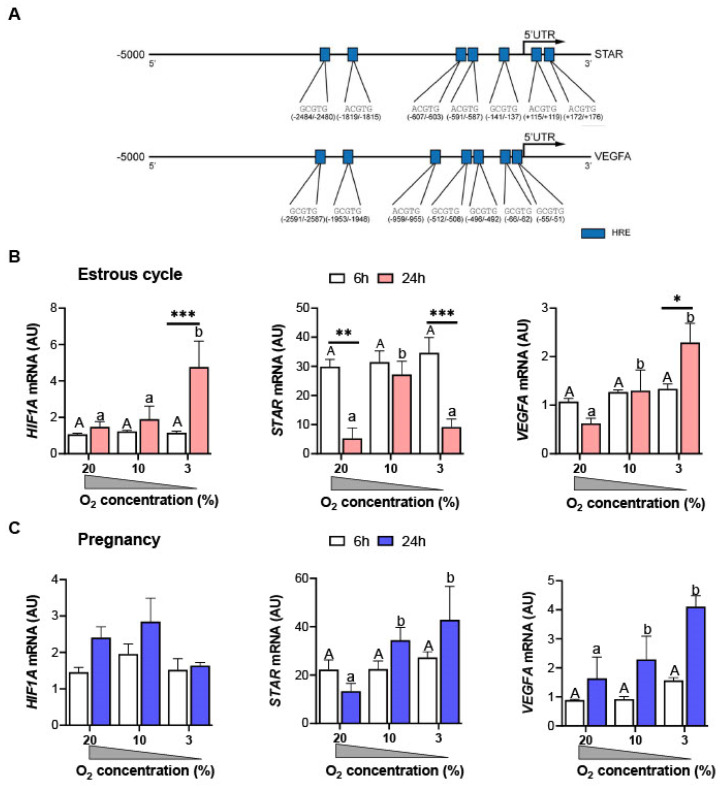
Hypoxia response elements (HREs) [(A/G)CGTG] identified within the DNA sequences of a promoter (5000 bp upstream) of porcine *STAR* and *VEGFA* (**A**). The expression of mRNA in the luteal tissue slices of cyclic (day 13 of the estrous cycle; (**B**)) and/or early pregnant gilts (day 13 of pregnancy; (**C**)) after incubation in the different O_2_ concentrations (20, 10, and 3% O_2_) for 6 and 24 h. Data were normalized to GAPDH (AU) and are presented as mean ± SEM (*n* = 5–7 per reproductive status). The analysis was performed by using a two-way ANOVA with Bonferroni post hoc test. Capital and small letters denote significant differences between the different concentrations of O_2_ across 6 and 24 h incubations, respectively. Significant changes between the time of incubation are indicated with asterisks (* *p* < 0.05, ** *p* < 0.01, and *** *p* < 0.001). AU, arbitrary units.

The bioinformatic screening revealed that *STAR* and *VEGFA*, known important regulators of luteal function, contain seven HRE binding sites within the 5000-bp upstream promoter region (Figure 2A). In the *HIF1A* promoter region, nine HRE binding sites were found. Thus, the expression of all three genes was analyzed in the luteal tissue of gilts incubated in different concentrations of O_2_. Incubation of the luteal tissue of cyclic or pregnant gilts for 6 h in high and both reduced O_2_ conditions did not affect the mRNA expression of *HIF1A*, *STAR*, and *VEGFA* (Figure 2B,C). After 24 h of incubation, the elevated mRNA expression of *HIF1A* was noticed in the luteal tissue of cyclic gilts cultured in 3% O_2_ vs. 20% and 10% O_2_ (2.9- and 2.5-fold increase, respectively; *p* < 0.05). The mRNA level of *STAR* was higher in the tissue slices of cyclic gilts incubated in 10% O_2_ vs. 20% and 3% O_2_ (5.1- and 3.0-fold increase, respectively; *p* < 0.01), while the mRNA expression of *VEGFA* increased after incubation in 10% and 3% O_2_ vs. 20% O_2_ (2.1- and 3.7-fold increase, respectively; *p* < 0.05 and *p* < 0.01, respectively; Figure 2B). In pregnant gilts, the mRNA expression of *STAR* and *VEGFA* was elevated (*p* < 0.05) in the luteal tissue cultured in 10% and 3% O_2_ vs. 20% O_2_ (2.6- and 3.2-fold increase, respectively, for *STAR*; 1.4- and 2.5-fold increase, respectively, for *VEGFA*; Figure 2C).

### 3.3. HIF-1α Content Is Elevated in the Corpus Luteum of Cyclic Gilts

**Figure 3:**
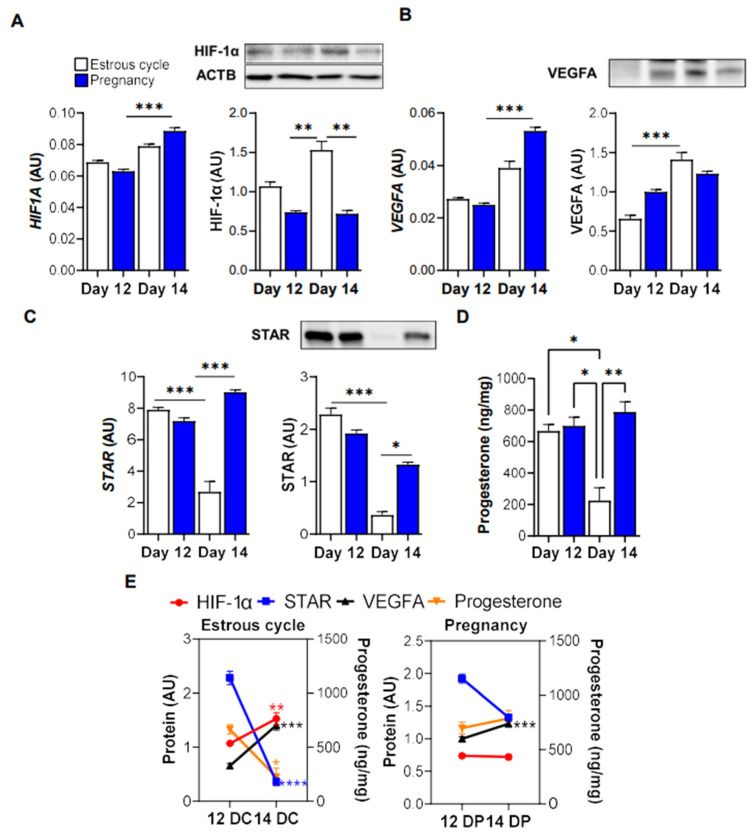
The expression of HIF-1α, *VEGFA* and *STAR* at mRNA and protein levels in the porcine corpus luteum on day 12 and 14 of the estrous cycle and pregnancy. Gene expression was normalized to GAPDH (AU). Protein levels were normalized to ACTB (for HIF-1α) or total protein content (AU) using TGX Stain-Free gel technology (**A**–**C**). Representative blots are presented above the graphs. The concentration of progesterone in the porcine corpus luteum on day 12 and 14 of the estrous cycle and pregnancy (**D**). Changes in the protein expression of HIF-1α, *STAR* and *VEGFA* levels (left *y* axis) and progesterone concentration (right *y* axis) in the porcine corpus luteum on day 12 and 14 of the estrous cycle (12DC; 14DC) or pregnancy (12DP; 14DP) (**E**). Comparison was made based the on data presented in subgraphs (**A**–**D**). All data are presented as mean ± SEM (*n* = 5–7 per reproductive status). The analysis was performed by using a one- and two-way ANOVA with Tukey and Bonferroni post hoc test. Significant changes are indicated as * *p* < 0.05, ** *p* < 0.01, *** *p* < 0.001, **** *p* < 0.0001. AU, arbitrary units.

Knowing that hypoxia and HIF-1 can regulate the expression of *STAR* and *VEGFA* in steroidogenic cells [10,26,35], we analyzed the expression of HIF-1α, *VEGFA* and *STAR* at mRNA and protein levels in the porcine corpus luteum on day 12 and 14 of the estrous cycle and pregnancy. The mRNA expression of *HIF1A* was significantly higher (1.42-fold increase; *p* < 0.001) in the corpus luteum on day 14 in comparison to day 12 of pregnancy (Figure 3A). Protein expression of HIF-1α markedly increased (*p* < 0.01) on day 14 of the estrous cycle in comparison to day 12 and the parallel day of pregnancy (1.42- and 2.1-fold increase, respectively; Figure 3A). The mRNA expression of *VEGFA* increased (*p* < 0.001) on day 14 of pregnancy vs. day 12 of pregnancy (2-fold increase), whereas *VEGFA* protein expression was elevated (2.2-fold increase; *p* < 0.001) on day 14 of the estrous cycle in comparison to day 12 (Figure 3B). The mRNA and protein expression of *STAR* prominently dropped (*p* < 0.001) on day 14 of the estrous cycle vs. day 12 of the estrous cycle and parallel day of pregnancy (67% and 71% reduction, respectively) (Figure 3C). Similarly, the luteal progesterone concentration decreased (*p* < 0.01) on day 14 of the estrous cycle in comparison to day 12 of the estrous cycle and both days of early pregnancy (67%, 68% and 72% reduction, respectively; Figure 3D). Based on obtained results, we prepared figures presenting changes in the protein expression of HIF-1α, *VEGFA*, *STAR* and intraluteal progesterone concentration between day 12 and 14 of the estrous cycle and pregnancy (Figure 3E).

### 3.4. The Levels of Phosphorylated cJUN and CREB Are Elevated in the Luteal Tissue of Pregnant Gilts

**Figure 4:**
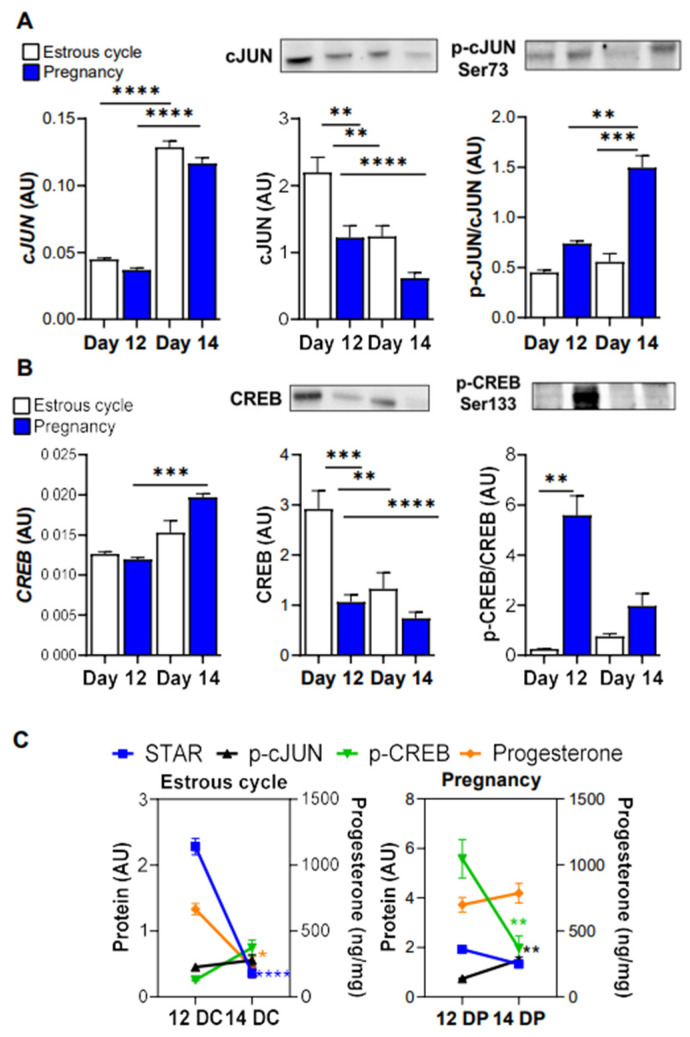
The expression of cJUN and CREB at mRNA and protein level in the porcine corpus luteum on day 12 and 14 of the estrous cycle and pregnancy. Gene expression was normalized to GAPDH (AU). Protein levels were normalized to total protein content (AU) using TGX Stain-Free gel technology (**A**,**B**). Representative blots are presented above the bar graphs. Changes in the content of STAR, p-cJUN and p-CREB (left *y* axis) and progesterone concentration (right y axis) in the porcine corpus luteum on day 12 and 14 of the estrous cycle (12DC; 14DC) or pregnancy (12DP; 14DP) (**C**). Comparison was made based on the data presented in subgraphs (**A**,**B**) and Figure 3D. Data are presented as mean ± SEM (*n* = 5–7 per reproductive status). The analysis was performed by using a one- and two-way ANOVA with Tukey and Bonferroni post hoc test. Significant changes are indicated as * *p* < 0.05, ** *p* < 0.01, *** *p* < 0.001, **** *p* < 0.0001. AU, arbitrary units.

cJUN and CREB were identified as two transcription factors regulating *STAR* expression in steroidogenic cells [9,35]. Thus, next, we measured mRNA and protein expression as well as the content of phosphorylated cJUN and CREB in the corpus luteum of cyclic and pregnant gilts. The expression of *cJUN* was 3-fold higher (*p* < 0.0001) in the luteal tissue of cyclic and pregnant gilts on day 14 vs. day 12. cJUN protein expression was elevated on day 12 in comparison to day 14 of the estrous cycle and studied days of pregnancy. Levels of phosphorylated cJUN (Ser73) markedly increased (*p* < 0.001) on day 14 vs. day 12 of pregnancy and the parallel day of the estrous cycle (2.71- and 2.03-fold increase; Figure 4A). The mRNA expression of *CREB* was elevated (*p* < 0.001) in the luteal tissue on day 14 in comparison to day 12 of pregnancy (1.72-fold increase). Protein expression of CREB was the highest in the luteal tissue on day 12 in comparison to day 14 of the estrous cycle and both days of pregnancy, whereas the level of phosphorylated CREB (Ser133) was 6.5- fold higher (*p* < 0.01) in the luteal tissue on day 12 of pregnancy vs. the parallel day of the estrous cycle. Based on the obtained results we prepared figures presenting changes in the content of *STAR*, intraluteal progesterone concentration and phosphorylated cJUN and CREB between day 12 and 14 of the estrous cycle and pregnancy (Figure 4C).

## 4. Discussion

The present study provides novel insights into mechanisms regulating the maintenance of corpus luteum function in gilts. First, in vitro experiments employing precision-cut luteal tissue slices, which contain all types of corpus luteum cells, demonstrated that: (1) a reduced concentration of O_2_ changes the mRNA expression of *HIF1A*, *STAR* and *VEGFA*; (2) a reduced concentration of O_2_ decreases the basal production of progesterone in the corpus luteum of cyclic and pregnant gilts; and (3) luteal tissue of cyclic and pregnant gilts possesses similar sensitivity towards hypoxia. Furthermore, ex vivo experiments suggested that *STAR* expression and progesterone production can be dependent on CREB and cJUN in the corpus luteum of early pregnant gilts. Moreover, elevated protein expression of HIF-1α in the corpus luteum at the late-luteal phase may suggest its potential role in the luteal regression but it remains to be further investigated. 

Hypoxia was found as an important regulator of steroidogenesis in granulosa and luteal cells [10,13,14,15,16,17,18,22,23,24,25,30,36,37]. As far as hypoxia increased progesterone production in luteinized bovine granulosa cells or murine KK1 cells, it had a negative effect on the biosynthesis of progesterone in the primary bovine luteal cells isolated during the early- and mid-luteal phase [10,17,18]. In our study, hypoxic conditions similarly suppressed basal production of progesterone in the luteal tissue of both cyclic and pregnant gilts. Interestingly, the luteal tissue of pregnant gilts produced minimal amounts of progesterone after culture in 10% O_2_. In comparison, the luteal tissue of cyclic gilts secreted similar reduced amounts of progesterone in the lowest O_2_ tension (3%). Incubation in hypoxic conditions (3% O_2_) for 6 h reduced basal or hCG-stimulated progesterone synthesis by bovine luteal cells isolated from the mid-luteal phase but did not affect the secretory function of early luteal phase cells [14,18]. Longer incubation in the same O_2_ tension impaired basal and hCG-stimulated progesterone production by cells from both periods of luteal-phases suggesting that cells obtained from different stages of the luteal phase respond to low concentrations of O_2_ [14]. 

A low concentration of O_2_ induces hypoxic conditions that prevent degradation of HIF-1α/*HIF1A* via the ubiquitin–proteasome pathway leading to its stabilization in the cytoplasm. HIF-1α acts in parallel with constitutively expressed HIF-1β, forming transcription factor HIF-1 [38]. Many of the most prominent and well-characterized targets of HIF-1 are involved in angiogenesis, erythropoiesis, cell metabolism, viability, and proliferation [21]. Herein, hypoxia response elements (HREs) were found within porcine *STAR* and *VEGFA* promoters, while gradually decreasing concentrations of O_2_ promoted the mRNA expression of both genes in the luteal tissue of pregnant gilts. In cyclic gilts, incubation of luteal slices in hypoxic conditions had a stimulatory effect on *VEGFA* mRNA expression. The mentioned results are in line with previous studies on primary luteal and granulosa cells or mouse KK1 cell line [10,13,14,15,16,17,18,20,22,24,39]. Interestingly, higher mRNA expression of *STAR* was noticed in the luteal tissue of cyclic gilts incubated in 10% vs. 20% and 3% O_2_, suggesting that O_2_ concentration can differentially regulate the expression of some targeted genes, and the degree of hypoxia appears to matter with HIF-1 complexes being required for the functionality of steroidogenic cells. Partial hypoxia (10% O_2_) strongly enhanced progesterone production as well as *STAR* mRNA and protein expression in KK1 cells or bovine granulosa cells [10]. In contrast, severe hypoxia (5–3% O_2_) impeded progesterone production but did not affect *STAR* expression, thus indicating that other mechanisms can affect steroidogenesis [10,14]. It should not be excluded that reduced O_2_ levels can differentially regulate targets at the mRNA and protein levels. Moreover, we should treat these results with caution because luteal tissue morphology and protein expression in luteal slices cultured in hypoxic conditions were not analyzed in the present study. Therefore, further studies should be undertaken to confirm the effects of hypoxia on protein expression in different compartments of luteal tissue.

In the gonads, acute steroidogenesis is mediated by mechanisms that enhance the transcription, translation, or the activity of *STAR* [9,40], however the mechanisms regulating *STAR* expression in the luteal tissue of gilts remain unknown. *STAR* expression can be regulated by several transcription factors such as cJUN, CREB and HIF-1 [9,10,35]. Considering the stimulatory effects of lower O_2_ tension on *STAR* and *VEGFA* mRNA expression in the luteal tissue of pregnant animals, we analyzed the protein expression of HIF-1α in the corpus luteum collected at the time of luteal regression and rescue. Herein, elevated protein expression of HIF-1α accompanied with high protein levels of *VEGFA* but decreased levels of *STAR* and progesterone concentration were observed in the luteal tissue of cyclic gilts indicating the occurrence of functional luteolysis. A significant reduction in ovarian arterial blood flow at the time of regression limits the supply of substrates for steroidogenesis, luteotropic support and O_2_ to the luteal tissue [37]. Therefore, we posit that reduced O_2_ levels and perhaps HIF-1α can be involved in cyclic functional luteolysis in gilts as it was suggested previously in the bovine corpus luteum [18]. Notably, the content of HIF-1α did not change in the luteal tissue of pregnant gilts. Still, it should not be excluded that the HIF-1 participates in luteal function maintenance and thus further mechanistic studies should be undertaken to confirm these suppositions. 

Manna and Stocco [9] proposed that PKA activation by trophic hormones phosphorylate CREB and cFos/cJun, both of which bind to the CRE2/AP-1 motif in the *STAR* promoter and recruit the transcriptional coactivator CBP. Once activated, p-CREB and p-cJUN are translocated to the nucleus, where they bind to responsive elements present within the *STAR* promoter and activate it in a temporal manner, with p-CREB activity lasting longer and resulting in high p-CREB/p-cJUN ratio [9,35]. This mechanism could explain maintained high levels of *STAR* and progesterone in the luteal tissue on day 12 of pregnancy, when the content of phosphorylated CREB was markedly elevated. Interestingly, the maternal recognition of pregnancy in gilts starts on day 12 after conception with simultaneous change in the luteolytic sensitivity of luteal tissue, which remains resistant to the luteolytic action of prostaglandin F2α [1,2,19]. One of the most prominent changes noticed in the porcine corpus luteum during maternal recognition of pregnancy was high levels of intraluteal prostaglandin E2 (PGE2), its receptor, Prostaglandin E Receptor 4 (PTGER4), and increased production of cyclic adenosine monophosphate (cAMP) and progesterone by the luteal tissue of pregnant gilts in response to PGE2 treatment [19,41]. Since cAMP evokes PKA signaling and consequently phosphorylation of CREB, the observed herein elevated content of phosphorylated CREB in the luteal tissue of pregnant gilts is possibility caused by activation of PGE2 signaling and is required to maintain the high steroidogenic capacity of luteal cells. The high levels of p-CREB with simultaneous increases in *STAR* and progesterone levels were observed in steroidogenic cells [42]. Recently, Lanfranchi et al. [35] found that HIF-1 increases *STAR* expression via stimulatory effects on the expression of transcriptional coactivator CBP and cJUN as well as the binding of p-CREB (Ser133) to *STAR* promoter in mouse KK1 granulosa cells. Based on their findings they proposed a model of the involvement of cJUN in the hypoxia- and HIF1α-dependent activation of *STAR* protein expression and function in immortalized granulosa cells. Herein, HIF-1α appears to be present in the corpus luteum of cyclic and pregnant gilts, albeit at a relatively low level. Considering multifunctional regulation of HIF-1α stability [43], we cannot exclude that HIF-1 can regulate luteal function maintenance during early pregnancy in pigs. Further studies would be required to test if the mentioned mechanisms occur in the porcine corpus luteum. 

## 5. Conclusions

The present study provides evidence that decreasing O_2_ tension reduces basal progesterone production by the in vitro cultured luteal tissue of cyclic and pregnant gilts. Together with results of the ex vivo experiment demonstrating elevated protein expression of HIF-1α and the drop in progesterone concentration in the corpus luteum at the late-luteal phase, it suggests the role of reduced O_2_ supply and perhaps HIF-1α in the luteal regression. High levels of phosphorylated CREB and cJUN may be important in maintaining the high steroidogenic capacity of luteal cells during early pregnancy in gilts. Further studies explaining the hypoxic effects on the global molecular changes and determining factors that affect luteal tissue HIF-1 stability are needed to further explain the sequence of events activated by hypoxia during the regression and rescue of the corpus luteum in cyclic and early pregnant gilts, respectively. Considering endothelial cells as an important target of hypoxia, demonstration of cellular and subcellular localization of HIF-1 in the ex vivo luteal tissue collected at different days of the estrous cycle and pregnancy, as well as in vitro luteal slices cultured in reduced O_2_ levels, would be helpful to understand the mechanisms of hypoxia action in the luteal tissue.

## Data Availability

None of the data were deposited in an official repository.
